# Molecular Basis of the Slow Growth of *Mycoplasma hominis* on Different Energy Sources

**DOI:** 10.3389/fcimb.2022.918557

**Published:** 2022-07-07

**Authors:** Daria V. Evsyutina, Tatiana A. Semashko, Maria A. Galyamina, Sergey I. Kovalchuk, Rustam H. Ziganshin, Valentina G. Ladygina, Gleb Y. Fisunov, Olga V. Pobeguts

**Affiliations:** ^1^ Department of Molecular Biology and Genetics, Federal Research and Clinical Center of Physical-Chemical Medicine, Federal Medical Biological Agency Malaya Pirogovskaya 1a, Moscow, Russia; ^2^ Department of Systems and Synthetic Biology, Scientific Research Institute for Systems Biology and Medicine Nauchniy proezd 18, Moscow, Russia; ^3^ Shemyakin-Ovchinnikov Institute of Bioorganic Chemistry, Russian Academy of Sciences Miklukho-Maklaya 16/10, Moscow, Russia

**Keywords:** slow growth, antibiotic sensitivity, *Mycoplasma hominis*, proteomics, thymidine

## Abstract

*Mycoplasma hominis* is an opportunistic urogenital pathogen in vertebrates. It is a non-glycolytic species that produces energy *via* arginine degradation. Among genital mycoplasmas, *M. hominis* is the most commonly reported to play a role in systemic infections and can persist in the host for a long time. However, it is unclear how *M. hominis* proceeds under arginine limitation. The recent metabolic reconstruction of *M. hominis* has demonstrated its ability to catabolize deoxyribose phosphate to produce ATP. In this study, we cultivated *M. hominis* on two different energy sources (arginine and thymidine) and demonstrated the differences in growth rate, antibiotic sensitivity, and biofilm formation. Using label-free quantitative proteomics, we compared the proteome of *M. hominis* under these conditions. A total of 466 proteins were identified from *M. hominis*, representing approximately 85% of the predicted proteome, while the levels of 94 proteins changed significantly. As expected, we observed changes in the levels of metabolic enzymes. The energy source strongly affects the synthesis of enzymes related to RNA modifications and ribosome assembly. The translocation of lipoproteins and other membrane-associated proteins was also impaired. Our study, the first global characterization of the proteomic switching of *M. hominis* in arginine-deficiency media, illustrates energy source-dependent control of pathogenicity factors and can help to determine the mechanisms underlying the interaction between the growth rate and fitness of genome-reduced bacteria.

## Introduction

Pathogenic bacteria can infect and persist in their hosts for a long time. They have developed different ways to cope with adverse conditions and to be invisible to the host’s defense systems. Slow growth is one such strategy (Gray et al., 2019). Spreading inside the host, bacteria encounter different host environments with different nutrient availabilities. Some bacteria adapt their metabolic activity to host environments (Koczula et al., 2017) that allows them to occupy different niches.

*M. hominis* is a facultative-pathogenic cell wall-less bacterium that is found as a commensal bacterium in the urogenital tract but is also associated with pelvic inflammatory disease, bacterial vaginosis, and severe complications in pregnancy (Donders et al., 2017). The metabolic capabilities of *M. hominis* are reduced and seem to adapt to their natural niche. *M. hominis* is a non-glycolytic species due to the absence of 6-phosphofructokinase (Pfk) (Pereyre et al., 2009). This bacterium produces energy through arginine or dimethylarginine degradation by four enzymes. In the first step, arginine deiminase (ArcA) hydrolyzes arginine to citrulline and ammonia or N(G),N(G)-dimethylarginine dimethylaminohydrolase (DDAH) metabolizes dimethylarginine to citrulline and dimethylamine. In the second step, ornithine carbamoyltransferase (ArcB) converts citrulline to ornithine and carbamoyl phosphate in the presence of phosphate. The final step is the synthesis of ATP by carbamate kinase (ArcC) from carbamoyl phosphate and ADP (Pereyre et al., 2009). Free N(G),N(G)-dimethyl-L-arginine, like other arginine and lysine analogs, can be isolated from human urine. The concentration of N(G),N(G)-dimethyl-L-arginine in human urine is higher than that of arginine (Kakimoto and Akazawa, 1970). Recent data suggest that *M. hominis* can utilize ribose-phosphate and deoxyribose-phosphate, particularly thymidine, formed by nucleoside catabolism as an energy source. Generally, thymidine phosphorylase (DeoA) reversibly phosphorylates pyrimidine nucleosides to deoxyribose-1-phosphate and pyrimidine bases. Phosphopentomutase (DeoB) converts deoxyribose-1-phosphate to deoxyribose-5-phosphate that is further converted to acetaldehyde and glyceraldehyde 3-phosphate (G3P) by deoxyribose-phosphate aldolase (DeoC). G3P can undergo sequential enzymatic reactions by glyceraldehyde-3-phosphate dehydrogenase (Gap), phosphoglycerate kinase (Pgk), phosphoglucomutase (Pgm), enolase (Eno), pyruvate kinase (Pyk), and lactate dehydrogenase (Ldh) to form lactate, generating ATP as a by-product (Pereyre et al., 2009; Fisunov et al., 2022),. *M. hominis* has been reported to leave its natural niche and exists in various organs and tissues (Mian et al., 2005; Henao-Martínez et al., 2012; Gagneux-Brunon et al., 2015). Thus, the question arises: what changes do occur in *M. hominis* under arginine and its derivatives-restriction conditions and how does this affect its physiology? We hypothesized that changes in metabolism, primarily the ability to use deoxyribose as an energy source, may be involved in the adaptation to a new niche and proliferation of *M. hominis.*

In this study, we cultivated *M. hominis* on two different energy sources (arginine and thymidine) and demonstrated the differences in growth rate, antibiotic sensitivity, and biofilm formation. Using label-free quantitative proteomics, we identified proteins that were differentially expressed under these conditions. These proteins are likely to explain the phenotypic features of *M. hominis* grown on arginine and thymidine and reveal the molecular mechanisms underlying slow growth as a way to adapt to a new niche and spread.

## Materials and Methods

### Bacterial Strains and Growth Conditions

The *M. hominis* H34 strain was grown on Brain Heart Infusion medium (DIFCO, USA) supplemented with 10% horse serum (Biolot, Russia), 1% yeast extract (Helicon, Russia), and penicillin (Sintez, Russia) with a final concentration of 500 units/mL with the addition of 1% arginine or 20 mM thymidine as a carbon source. The cultures were incubated at 37 °C in aerobic condition. The growth curves were monitored through total amount of genomic DNA. The R package Growthcurver was used to fit the growth curve data to the logistic equation (Sprouffske and Wagner, 2016). The value of arguments (r, the growth rate) were extracted (**Supplementary Table S1**). The 0.002% (w/v) phenol red (Sigma-Aldrich, USA) was added in the culture medium to indirect detect of metabolic activity. A color change of medium was monitored using the OD at 560 nm (Cummings and McCormack, 1990).

### DNA Extraction

The genomic DNA was isolated from 200 μL of bacterial cell suspension. *M. hominis* cells were harvested by centrifugation (12000 g for 10 min, 4 °C) and lyzed with CTAB buffer [2% CTAB, 100 mM Tris–HCl (pH 8.0), 20 mM EDTA, and 1.4 M NaCl] at 60 °C for 30 min with subsequent chloroform extraction (1:1) and isopropanol precipitation (1:1) with the addition of 10% v/v 3M sodium acetate (pH 5.2) (Stewart and Via, 1993). The pallets of nucleic acids were washed with 80% ethanol and finally resuspended in 22 µl of mQ (Panreac, Spain). The amount of DNA was determined using the Qubit 2.0 fluorometer (Thermo Fisher Scientific, USA).

### Quantitative Real-Time PCR

Quantitative real-time PCR was performed using dNTP, PCR buffer, Taq-polymerase (Lytech, Russia), SYBR Green I (Invitrogen, USA), and CFX96 Real-Time PCR Detection System (Bio-Rad, USA) PCR machine. Primers for *tuf* amplification were used (tuf_mho_F: TATTGCTACGTGGAATTGACAG; tuf_mho_R: CCTTCACGAATAGAGAACTTGG). Primers were designed using BAC-Browser (Garanina et al., 2018). Each 20-µl reaction contained 1 µL of template gDNA. Thermal cycling conditions were as follows: initial denaturation at 95 °C for 1 min, then 40-cycle amplification (94 °C for 15 s, 58 °C for 20 s, and 68 °C for 40 s) with a single fluorescence per reading. Melting curve was obtained by gradually heating the PCR mixture from 65 to 92 °C at a rate of 0.5 °C every 5 s, with continuous fluorescence scanning. All PCR experiments were carried out in three replicates. The efficiency of the PCR amplification was determined for *tuf* primer pair. Standard curve was plotted for five twofold serial dilutions of gDNA. The slope of the standard curve was used to calculate the PCR efficiency. For *tuf* primer pair, PCR efficiency was 105%. Three technical repeats were used (**Supplementary Table S2** **,** **Figure S2**).

### Plate Assay for Antibiotic Sensitivity Test

The following 4 antibiotics were used: tetracycline (Sigma-Aldrich, USA), ofloxacin (Sigma-Aldrich, USA), gentamicin (Dalhimpharm, Russia), and chloramphenicol (Sigma-Aldrich, USA). Stock solutions (20.0 μg/μL) of the antibiotics were prepared in the appropriate diluents and filter-sterilized using a 0.2-μm ABLUO^®^ syringe-driven filter unit (GVS Filter Technology, USA). The aliquots (1 mL) of the stock solutions were stored at −20°C until use. The antibiotic sensitivity test for *M. hominis* was performed by the broth microdilution method with different antibiotic concentration ranges of 0.25, 1, 2.5, 5, 10, and 50 μg/mL. The third passage culture of *M. hominis* H34 grown in an appropriate medium was diluted with BHI broth supplemented with arginine or thymidine, as described above. The initial cells biomass calculated as amount of genomic DNA was equal for both conditions. In summary, 198 μL of culture was dispensed into the wells of sterile flat-bottom microtiter plates with lids (Corning Inc., USA), and 2.0 μL of the appropriate amount of the antibiotic solution was added. The culture without antibiotic solution (positive), the culture supplemented with diluents (mQ or 0.5% C_2_H_5_OH) (positive), and bacteria-free media (negative) were used as controls. The plates were incubated at 37°C for 30 or 48 h (until sufficient growth of the positive control samples was achieved). After that, 200 μL of culture from each well was transferred into tubes, gDNA was isolated and qPCR was performed as described above. All experiments were performed in triplicate.

### Plate Assay for Biofilm Quantification

Biofilm density was measured using a crystal violet assay (McAuliffe et al., 2006). Cells were grown in 96-well plates for 7 days. Biofilms grown in microtiter plates were rinsed in PBS to remove non-adherent cells and stained with 0.5 % crystal violet solution for 30 min. Biofilms were then washed five times in distilled water before being left to dry at room temperature for at least 30 min. Crystal violet in stained biofilms in microtiter plates was solubilized by the addition of 200 μL 100 % ethanol. Biofilm production was quantified by measuring the absorbance (560 nm) of 100 μL of the solubilized crystal violet in a microplate. At least 24 wells were analyzed for each biofilm strain.

### Protein Extraction and In-Solution Digestion

Protein samples were prepared as described previously (Semashko et al., 2020). Briefly, 10 mL of log-phase growing *M. hominis* H34 cells were collected by centrifugation at 12,000 × g at 4 °C for 10 min and washed twice with a cold PBS buffer. The cell pellets were resuspended in 10 μL of 10% sodium deoxycholate (DCNa) and a 0.5 μL of nuclease mix (GE Healthcare, USA) was added. After incubation for 1 h at 4 °C, the samples were resuspended in 100 µL of 100 mM Tris-HCl buffer (pH 8.0) containing 0.1% DCNa, 8 M urea, and 2.5 mM EDTA. The lysates were clarified by centrifugation at 16,000 × g for 10 min at 4 °C. The supernatants were saved, and the concentration was determined using a BCA Assay Kit (Sigma-Aldrich, USA). Two hundred micrograms of total protein per sample were used for peptide extract preparation. Disulfide bonds were reduced using 5 mM tris(2-carboxyethyl)phosphine hydrochloride (TCEP) (Sigma-Aldrich, USA) for 60 min at 37°C and alkylated with 30 mM chloroacetamide (Sigma-Aldrich, USA) for 30 min at room temperature in the dark. The sample was then diluted 6-fold with 50 mM Tris-HCl (pH 8.0) with 0.01% DCNa. Trypsin Gold (Promega, USA) was added to obtain the final trypsin:protein ratio of 1:50 (w/w) and incubated at 37 °C overnight. To stop digestion and degrade the acid-labile DCNa, trifluoroacetic acid (TFA) was added to a final concentration of 0.5% (v/v), incubated at 37 °C for 45 min, and the samples were centrifuged at 14,000  × g for 10 min to remove the DCNa. The peptide extract was desalted using a Discovery DSC-18 Tube (Supelco, USA) according to the manufacturer’s protocol. Peptides were eluted with 1 mL of 75% acetonitrile in water containing 0.1% TFA, dried in an Acid-Resistant CentriVap Benchtop Vacuum concentrator (Labconco, USA), and resuspended in 3% of acetonitrile in water containing 0.1% TFA to a final concentration of 5 μg/μL.

### Liquid Chromatography-Tandem Mass Spectrometry (LC-MS/MS) Analysis

Peptide products were analyzed on an Ultimate 3000 RSLC nano HPLC system connected to a QExactive Plus mass spectrometer (Thermo Fisher Scientific, USA). Samples were loaded onto a home-made trap column 20 × 0.1 mm packed with Inertsil ODS3 3 μm sorbent (GL Sciences, Japan), in loading buffer (2% ACN, 98% H2O, 0.1% TFA) at 10 μL/min flow and separated at RT in a home-packed fused-silica column 500 × 0.1 mm packed with Reprosil PUR C18AQ 1.9 (Dr. Maisch, Germany) into the emitter prepared with a P2000 laser puller (Sutter, USA) (Kovalchuk et al., 2019). Samples were eluted with a linear gradient of 80% ACN, 19.9% H_2_O, 0.1% FA (buffer B) in 99.9% H_2_O, 0.1% FA (solvent A) from 4% to 36% of solvent B in 1 h at 0.44 μL/min flow at RT. MS data were collected in DDA mode. MS1 parameters were as follows: 70 K resolution, 350 - 2000 scan range, maximum injection time 50 ms, and AGC target 3 × 10^6^. Ions were isolated with 1.4 m/z windows and 0.2 m/z offset targeting 10 highest intensity peaks of +2 to +6 charge, 8 × 10^3^ minimum AGC, preferred peptide match, and isotope exclusion. Dynamic exclusion was set to 40 s. MS2 fragmentation was carried out in HCD mode at 17.5 K resolution with 27% NCE. Ions were accumulated for a maximum of 45 ms with a target AGC of 1 × 10^5^.

### Proteomic Data Analysis

MS data were analyzed using the PEAKS software (https://www.bioinfor.com/) with default settings. The data were searched against the *M. hominis* ATCC 23114 NCBI database and were deposited in the ProteomeXchange Consortium *via* the PRIDE partner repository with the dataset identifier PXD018714 and project 10.6019/PXD018714 (http://dx.doi.org/10.6019/PXD018714, https://www.ebi.ac.uk/pride/archive/projects/PXD018714). Three biological replicates per each condition were used. For statistical analysis, a t-test with Benjamini–Hochberg correction was conducted. The abundance of proteins with fold change ≥ 1.5 and adjusted p-value < 0.05 were regarded as significant. Full proteomic data description is found in (Semashko et al., 2020). Significantly changed proteins for *M. hominis* growing on thymidine compared to the arginine-grown culture are described in **Supplementary Tables S3, S4**). *M. hominis* ATCC 23114 with complete genome assembly was used as a reference, but it can limit identification results due to possible genomic differences between strains ATCC 23114 and H34.

### *In Silico* Analysis

The available protein annotation for *M. hominis* ATCC 23114 is incomplete. The InterProScan software (Jones et al., 2014) was used to predict protein family membership and the presence of functional domains and sites. The analysis was run locally for all *M. hominis* ATCC 23114 protein sequences and the output results are available in **Supplementary Table S5**. Protein localization was predicted using PSORTb v3.0.2 (https://www.psort.org/psortb/), BUSCA (http://busca.biocomp.unibo.it/) (Savojardo et al., 2018), PRED-LIPO (http://www.compgen.org/tools/PRED-LIPO) (Bagos et al., 2008), LipoP 1.0 (http://www.cbs.dtu.dk/services/LipoP/) (Juncker et al., 2003), and SecretomeP 2.0a Server (http://www.cbs.dtu.dk/services/SecretomeP/) (Bendtsen et al., 2005). Programs for the prediction of signal peptides usually operate in two modes: gram-positive and gram-negative bacteria. Class Mollicutes is a specialized clade of Gram-positive bacteria but the signal peptides of mycoplasmas show closer sequence similarity to those of the Gram-negative bacteria (Song et al., 2009). We performed a search in both modes. Only the PSORTb v3.0.2 program proposes advanced options for the analysis of signal peptides in Mollicutes **Supplementary Table S6**. Transmembrane helices were predicted using the TMHMM server (Krogh et al., 2001).

## Results and Discussion

### *Mycoplasma hominis* Grown on Arginine and Thymidine Supplemented Medium Differ in the Carrying Capacity and the Growth Rate

**Figure 1:**
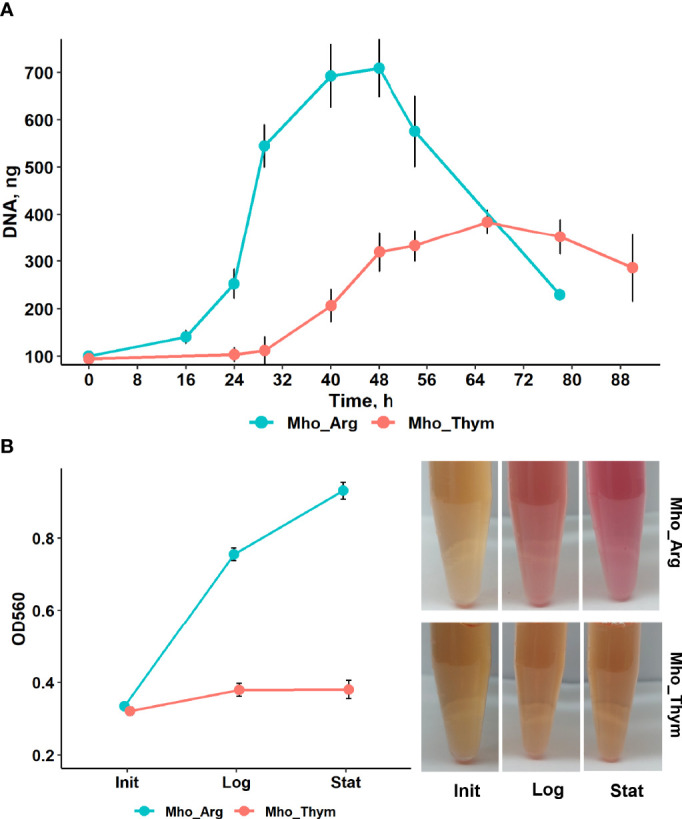
The growth curves of *M. hominis* on medium supplemented with arginine or thymidine. **(A)** Growth curves of *M. hominis*. All points are averages of triplicate experiments with standard deviation error bars **(B)** *M. hominis* was cultured in BHI media containing phenol red with arginine or thymidine. Arginine utilization and as a consequence production of ammonia was indicated by the color change of indicator to pink. The OD560 values were measured in three time point – after dilution of the third passage culture of *M. hominis* H34 in appropriate medium (Init), 0 h for both culture; in the mild-log phase (Log), 30 h for arginine supplemented culture, 42 h for thymidine; and in the stationary phase (Stat), 45 h and 65 h, respectively.

To examine how the substitution of energy sources was reflected in changes in the growth rates of *M. hominis*, we evaluated its growth curves *via* total amount of genomic DNA at several time points (**Figure 1A**). We used the R package Growthcurver (Sprouffske and Wagner, 2016) to fit the growth curve data using the standard logistic equation (Verhulst equation) commonly applied in ecology and evolution (Rockwood, 2015), and the value of arguments was extracted (**Supplementary Table S1**, **Figure S1**). The growth rate of *M. hominis* grown on thymidine was reduced as compared to that on arginine, the carrying capacity was two-fold lower. Thus, we conclude that *M. hominis* grown on thymidine undergoes significant energy limitation and a general metabolic slowdown.

In our study we used Brain Heart Infusion (BHI) medium supplemented with horse serum, yeast extract and arginine or thymidine. There is some amount of arginine or arginine-containing peptides in BHI. *M. hominis* produces energy through arginine or dimethylarginine degradation with the production of ammonia that leads to elevation of the pH medium. To evaluate the activity of arginine deiminase pathway of *M. hominis* in medium supplemented with arginine or thymidine, a pH testing experiment was performed. We added pH indicator phenol red in the culture medium and measured OD at 560 nm during mycoplasma growth (**Figure 1B**). The value of OD560 in BHI media with arginine significantly increased as bacteria growth. No color change occurred when the medium was supplied with thymidine. This allowed us to assume that activity of arginine deiminase pathway was dramatically reduced or absent. *M. hominis* could be grown in BHI without addition arginine (Pereyre et al., 2009) or thymidine but it lost ability to grow to third passage (Fisunov et al., 2022). How long *M. hominis* can be passaged on thymidine-containing media was not determined.

### The Growth Condition of *M. hominis* Influences Their Susceptibility to Antibiotics

**Figure 2:**
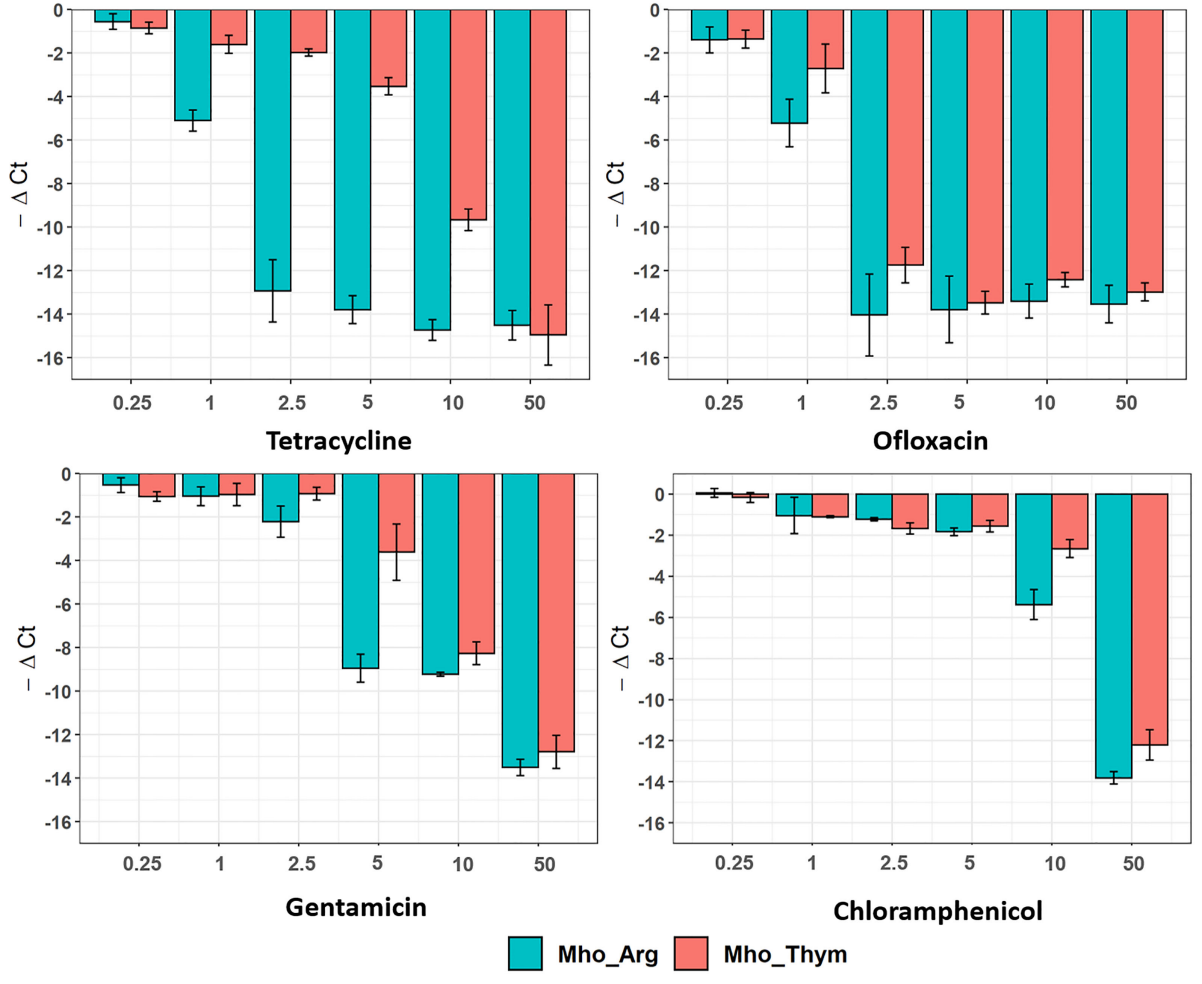
Susceptibility of *M. hominis* to antibiotics. *M. hominis* was cultivated on an arginine or thymidine-supplemented medium with different antibiotic concentration range: 0.25, 1, 2.5, 5, 10 and 50 μg/mL. Differences between Cq-values for antibiotic treatment samples and positive control samples without treatment are shown. All bar-plots are averages of triplicate experiments with standard deviation error bars.

We showed differences in the growth rates of *M. hominis* grown on different sources. For some bacteria, there is a direct correlation between growth rate and antibiotic efficiency (Lee et al., 2018). To examine this observation validity for *M. hominis*, we used the broth microdilution method with a wide range of antibiotics including tetracyclines (tetracycline), fluoroquinolones (ofloxacin), aminoglycosides (gentamicin), and amphenicols (chloramphenicol). Tetracyclines and fluoroquinolones are commonly used to treat *M. hominis* infections (Waites et al., 2005). A real-time PCR assay targeting *M. hominis tuf* (encodes elongation factor Tu) was performed to estimate bacteria viability. Mycoplasma under both growth conditions was equally resistant to chloramphenicol that inhibits protein biosynthesis by targeting the peptidyl transferase center on the large ribosomal subunit. In contrast, *M. hominis* was less susceptible to tetracycline that binds to the 16S rRNA at the 30S ribosomal subunit, and gentamicin. This effect was more pronounced for tetracycline (**Figure 2**). Comparation of melting curves for negative control samples and mycoplasma samples under the highest antibiotic concentration showed a peak of specific product (Tm=82°C) for mycoplasma samples and a lower temperature peak (Tm=75-77°C) for negative control samples (**Supplementary Figure S3**). This can be explained by the presence of dead or starved bacteria in the high-dose antibiotics treatment samples.

All ribosome-acting antibiotics used in this study reversibly bind to ribosomes and impair different activities. Tetracycline competitively inhibits aminoacyl-tRNA binding to the A-site, chloramphenicol inhibits transpeptidase activity, and gentamicin decreases the ribosome’s decoding specificity that leads to the transfer of non-cognate aminoacyl-tRNAs to the nascent peptide chain. We hypothesize that the observed increase in tetracycline resistance during growth on thymidine may be explained as follows. The growth on thymidine leads to a drastic slowdown in the growth rate, as demonstrated above. A slower growth rate leads to slower metabolism and energy starvation. Tetracycline competes with aminoacyl-tRNAs for A-site binding. However, the binding is reversible and aminoacyl-tRNAs have a chance to enter the A-site as well, albeit at a lower rate. We hypothesize that the translation rate in *M. hominis* grown on thymidine is even slower (due to energy starvation) than the tetracycline-mediated slow-down at the given concentration. Thus, tetracycline-mediated slowdown is not a limiting step. For gentamycin, the error rate does not depend on the translation rate. At the same time, *M. hominis* already shows substantial resistance to chloramphenicol, and thus the effect of the slow-down translation rate may not be detectable on the background of the observed resistance. However, this assumption needs to be confirmed. Mutations or modifications of a drug target could also explain the observed effect. However, after transfer and passaging on medium with arginine, *M. hominis* grown on thymidine became sensitive again (data not shown). Other mechanisms that can ensure bacterial resistance to antibiotics are discussed in the context of proteomic changes below. A study performed on Gram-positive and Gram-negative bacteria demonstrated a direct relationship between bacterial metabolism and bactericidal antibiotic efficacy. Currently, energy metabolism is considered a novel target pathway in drug discovery (Bald et al., 2017; Lopatkin et al., 2019).

### Effects of Energy Sources on Biofilm Formation Ability

**Figure 3:**
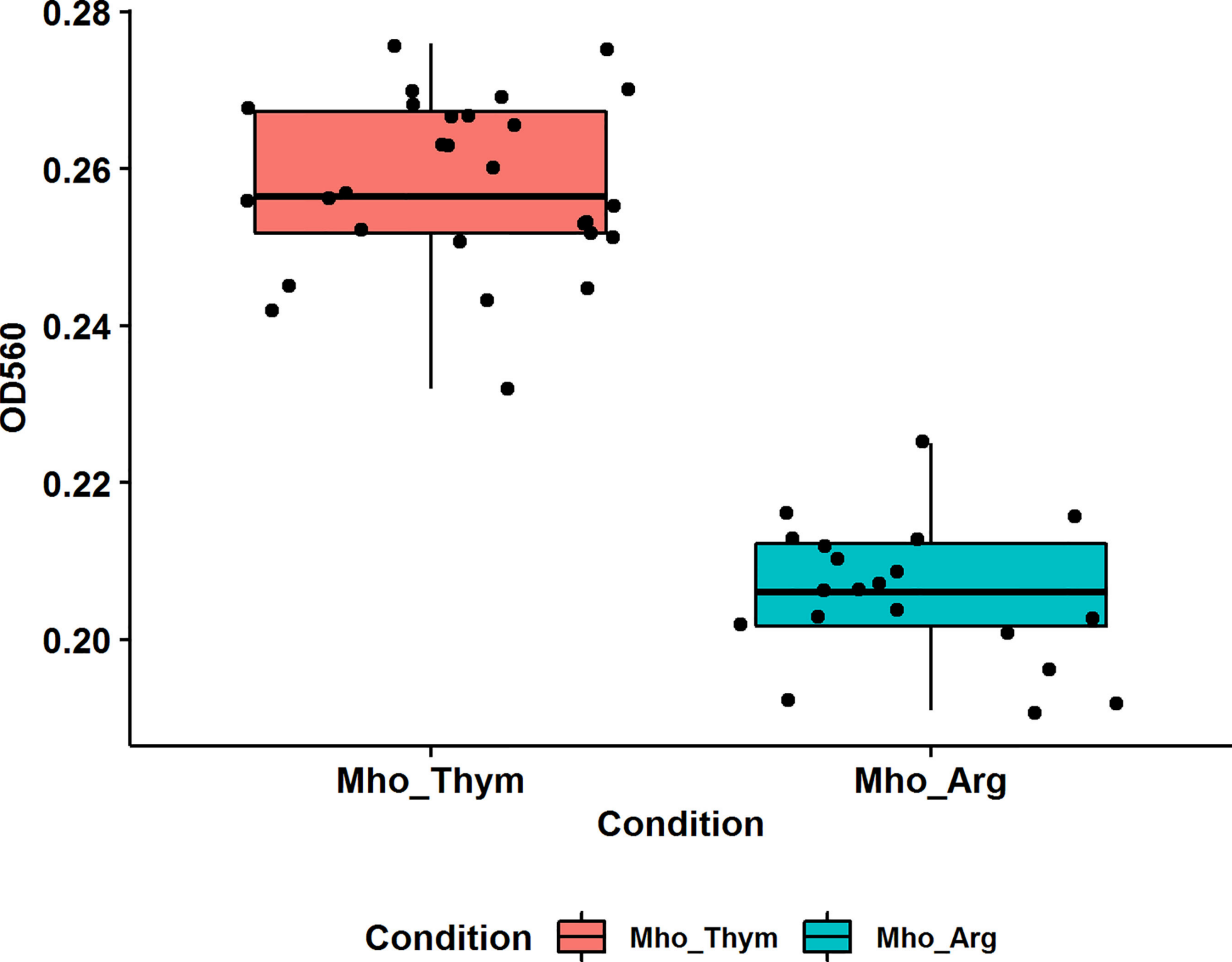
The effect of different carbon sources on *M. hominis* biofilm formation. Biofilm production was quantified by measuring the absorbance (560 nm) of crystal violet in a microplate. Groups are significantly different, pairwise comparisons using Wilcoxon rank sum test, p-value = 5e-09.

The ability to form biofilms is an advantage for bacteria. Cells in biofilms have increased resistance to host defenses and resistance to stress (Costerton et al., 1999; McAuliffe et al., 2006), including antibiotics (Feng et al., 2020; Schulze et al., 2021),. In this study, we estimated the effects of the energy source on biofilm formation by *M. hominis*. *M. hominis* were grown for 7 days at 37 °C. Growth on thymidine significantly enhanced biofilm production compared to growth on arginine-supplemented medium based on the OD at 560 nm measurements of bound crystal violet (**Figure 3**). A previous study showed that the extent of biofilm formation is very diverse among mycoplasma species (McAuliffe et al., 2006). Our results indicate the ability of *M. hominis* H34 to form biofilms *in vitro* as measured by cell density, and that it can be stimulated by energy sources.

### *M. hominis* Energy Metabolism Undergoes Rearrangement During Growth on Thymidine

It has been known that the major pathway of energy metabolism in *M. hominis* is arginine deamination (Pereyre et al., 2009). Previously, we performed the metabolic reconstruction of *M. hominis* and demonstrated that it is capable of growing on thymidine (Fisunov et al., 2022). We identified possible alternative pathways of energy metabolism: the utilization of ribose from ribonucleosides and the utilization of deoxyribose from deoxyribonucleosides. The alternative pathways start from the cleavage of nucleosides into nucleobases and phosphosugars. Phosphosugars are then processed to glyceraldehyde-3-phosphate that is utilized by the enzymes of lower glycolysis.

**Figure 4:**
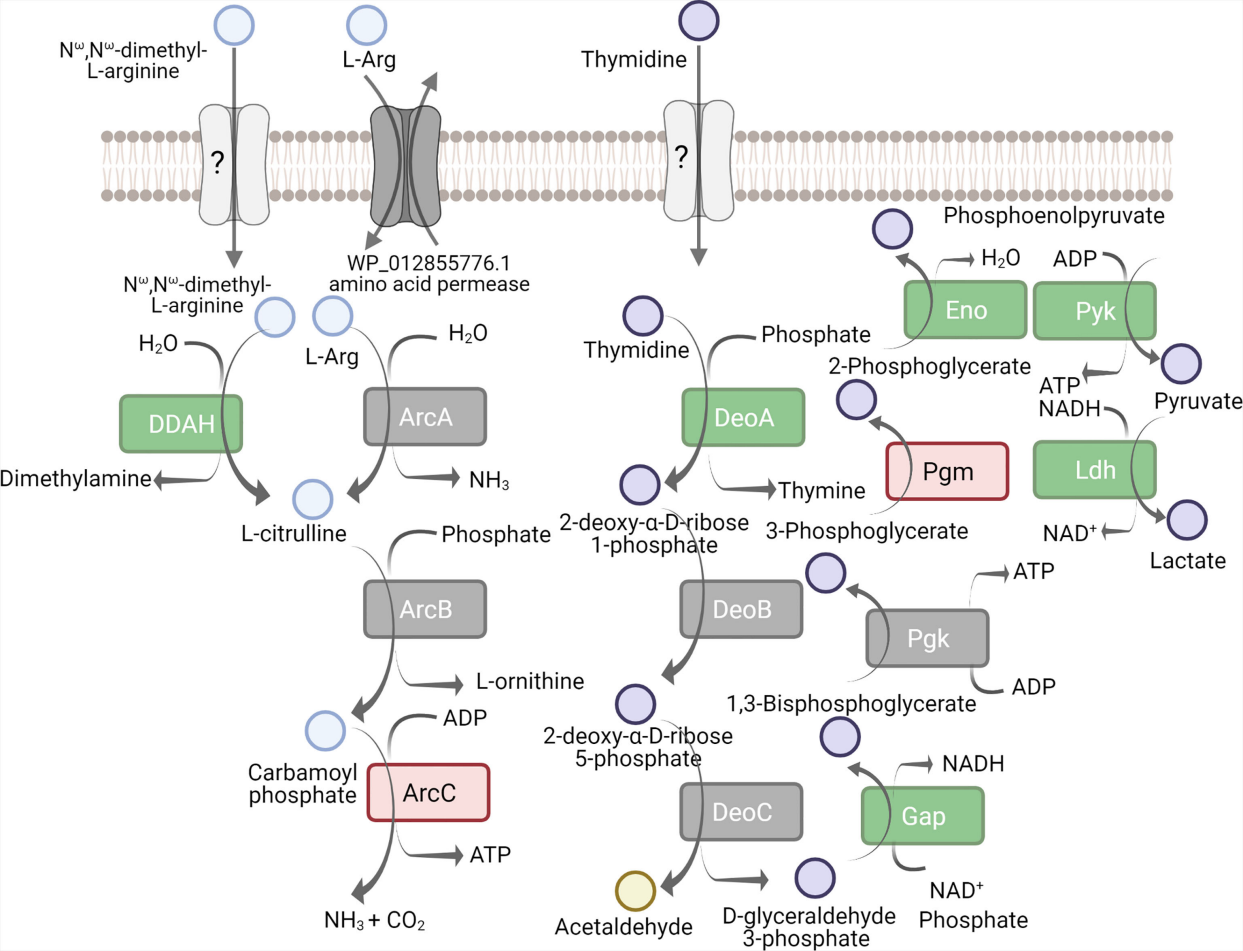
Proteins implicated in the arginine deiminase pathway and utilization of pyrimidine deoxynucleoside as energy source. Comparative proteomic analysis of *M. hominis* grown on thymidine-supplemented medium relative to arginine-supplemented medium. The down-regulated proteins are indicated red, up-regulated - in green, unchanged - in grey. Unknown proteins are in light grey. The full names of proteins are given in **Supplementary Tables S3**, **S4**.

To study the adaptation of *M. hominis* to growth on thymidine, we performed quantitative proteome analysis. Cells grown on arginine were used as the control. A total of 466 proteins were identified from *M. hominis*, representing approximately 85% of the predicted proteome. The abundance of 23 proteins was significantly increased and 71 decreased (adjusted p-value < 0.05) during growth on thymidine compared to growth on arginine. All proteins from the arginine deiminase pathway were identified, but the differential expression of only carbamate kinase (ArcC) changed slightly (**Figure 4**). The abundance of enzymes involved in the utilization of nucleosides including thymidine phosphorylase (DeoA), lactate dehydrogenase (Ldh), glyceraldehyde-3-phosphate dehydrogenase (Gap), and pyruvate kinase (Pyk) was significantly increased. Thus, we conclude that *M. hominis* rearranges its energy metabolism toward the utilization of nucleosides.

### The Energy Source Strongly Affects the Synthesis of Enzymes Related to RNA Modifications and Ribosome Assembly

**Figure 5:**
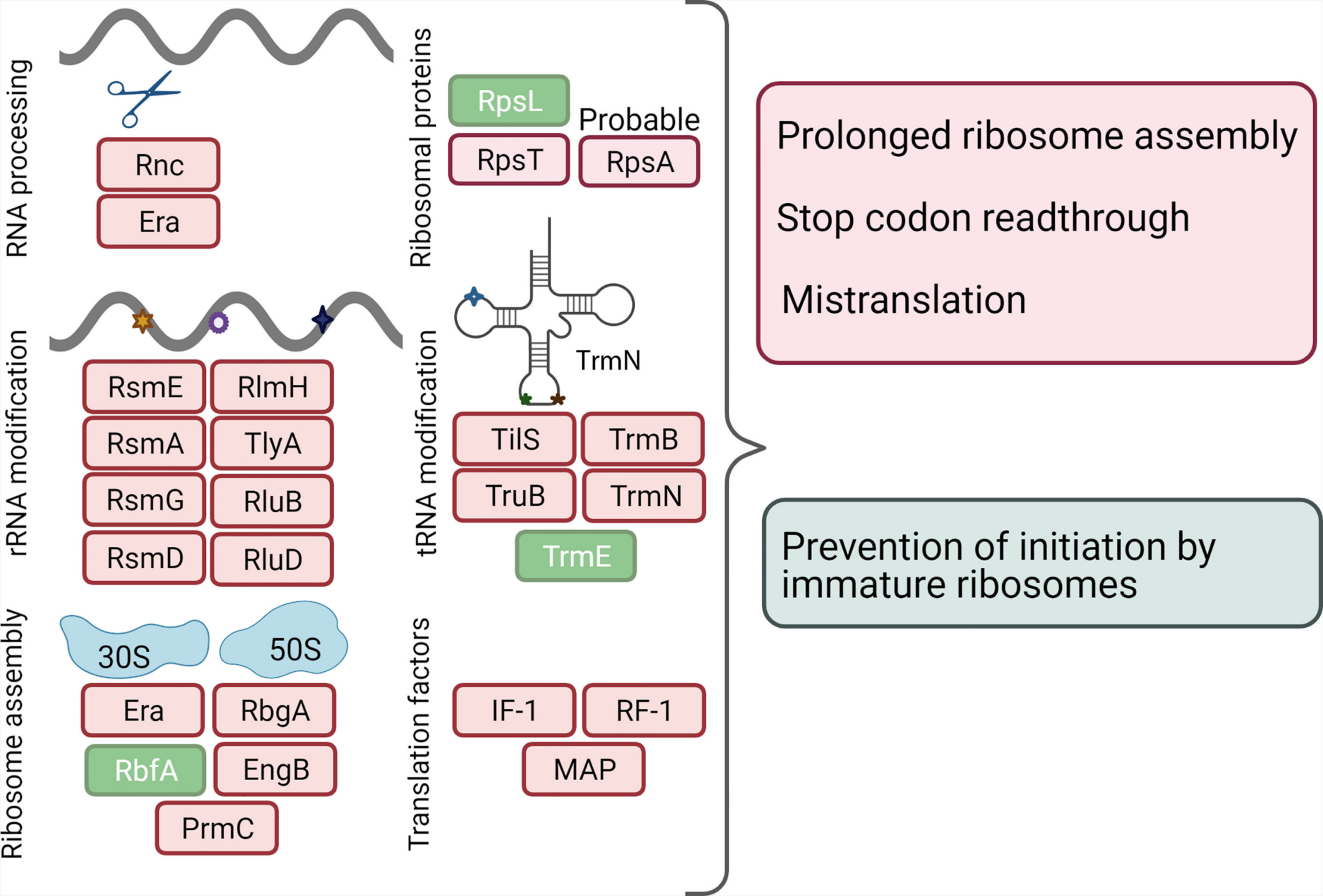
Response of translation apparatus to a change in the energy source. The down-regulated proteins are indicated in red, up-regulated - in green. The corresponding full names of proteins are given in **Supplementary Tables S3**, **S4**.

The switch of the energy source to thymidine resulted in numerous changes in the translation machinery of *M. hominis*. The abundance of the eight proteins involved in rRNA modification decreased (**Figure 5**). The number of enzymes that catalyze rRNA modification varies across bacteria. Mycoplasmas feature on average 10 enzymes of this function (De Crécy-Lagard et al., 2007). The modification targets are concentrated around the A and P sites, the peptidyl transferase center, the peptide exit tunnel, and on both sides of the intersubunit bridges (Sergiev et al., 2011). Numerous studies have shown that the loss of rRNA modifications results in the alteration of the structures of the active sites (Desaulniers et al., 2008; Demirci et al., 2010), that causes slower rates and lower accuracy of translation (Baxter-Roshek et al., 2007; Liang et al., 2007), as well as impaired responses to metabolites and antibiotics (Helser et al., 1971; Doi and Arakawa, 2007).

Among the proteins that are involved in the modification of tRNAs, four were down-regulated and only one, TrmE/MnmE (WP_012855730.1) was upregulated (**Figure 5**). tRNA modifications can occur in various positions. The number of modified nucleosides varies in different organisms from 2 to 30 - 50. It is believed that the modified nucleosides in and near the anticodon stem-loop (especially 34 and 37 nucleosides) can have critical roles, expanding or restricting the decoding properties of a given tRNA molecule; modifications outside the anticodon stem-loop have more structural roles (de Crécy-Lagard and Jaroch, 2021). In bacteria, the elimination of several anticodon stem-loop modifications gives rise to pleiotropic phenotypes. Moreover, recent studies have demonstrated that tRNA modifications in bacteria act as quality control signals, as in eukaryotes; the absence of some modifications leads to tRNA degradation (Kimura and Waldor, 2019). The upregulation of MnmE was surprising considering the downregulation of the majority of other modifying enzymes. MnmE is a multi-domain GTPase that is highly conserved among bacteria and eukaryotes (Armengod et al., 2012). Deletion of *mnmE* and its partner *mnmG* genes has been shown to have pleiotropic effects on growth, cell division, and virulence in a wide range of pathogenic bacteria, both gram-positive and gram-negative (Shippy and Fadl, 2014; Gao et al., 2019). However, all studies are rather phenomenological in nature; the mechanisms underlying the selective response of cells upon *mnmE* inactivation remain unknown.

In addition to modifications, the precursors of rRNAs and tRNAs undergo a processing stage catalyzed by endonucleases and exonucleases. We found that RNase III and the GTPase Era required for the maturation of ribosomal and other structural RNAs were downregulated by thymidine. In *Escherichia coli*, the *rnc* and *era* genes are transcribed from the same operon; moreover, their translation is coupled to ensure similar levels of expression (Britton et al., 1998). In the genome of *M. hominis*, *era* gene is far from *rnc* but its expression is apparently synchronized. A reduction in *E. coli* wild‐type *era* expression or reduced GTPase activity temporarily arrests cell growth at the two‐cell stages and delays cell division (Britton et al., 1998). The abundance of GTPases RbgA and EngB that are involved in ribosome biogenesis were also reduced during growth on thymidine. At the same time, 30S subunit-binding factor RbfA was upregulated. This protein is required for the efficient processing of the 5′- end of the 16S rRNA (Goto et al., 2011). A recent study demonstrated the unique ability of RbfA to suppress protein synthesis by immature *E. coli* 30S subunits, providing a quality control mechanism (Sharma and Woodson, 2020).

Growth on thymidine resulted in the downregulation of the protein modification enzyme PrmC (HemK). Among post-translational protein modification enzymes, only HemK is conserved in Mollicutes (Grosjean et al., 2014). This enzyme methylates the termination factor RF-1 (*prfA*) which is also downregulated during growth on thymidine. In *M. hominis* and *E. coli*, the initiation codon of *hemK* overlaps with the termination codon of *prfA*, suggesting that the synthesis of the two genes is coupled (Pierson et al., 2016). It has been shown that *hemK* knockouts of *E. coli K12* grow very slowly and translational read-through at the UAG codon recognized by RF-1 is increased. This could be due to the prolonged pause of the ribosome at the stop codons (Nakahigashi et al., 2002). In Mollicutes (except phytoplasmas and *Acholeplasma laidlawii*), another release factor (RF-2) is missed; therefore, a decrease in the levels of HemK and PrfA can have a stronger effect on translation in *M. hominis* than in *E. coli*.

Finally, WP_012855634.1 is another interesting protein whose level has decreased significantly with thymidine. This is a short protein (111 amino acids) annotated as hypothetical but containing an S1 RNA-binding structural domain. In some studies, this protein is considered to be ribosomal protein S1 (RpsA) that is lost in most Mollicutes and remains in several species of the Hominis subgroup (Grosjean et al., 2014). The well-studied bacterial 30S ribosomal protein S1 from *E. coli* (RpsA) has six S1 domains, while WP_012855634.1 has only one (Machulin et al., 2019). In *E. coli*, ribosomal protein S1 endows the 30S subunit with an RNA chaperone activity that is essential for the binding and unfolding of structured mRNAs, allowing the correct positioning of the initiation codon for translation (Duval et al., 2013). It remains unclear whether WP_012855634.1 has a function similar to that of RpsA from *E. coli*. Interestingly, the gene encoding WP_012855634.1, located upstream of *gpi* (glucose-6-phosphate isomerase) and *ldh* (lactate dehydrogenase) genes, and the end of possible *rpsA M. hominis*, intersects the start of *the gpi* gene. In prokaryotes, functionally related genes or their regulators are often organized into operons or colocalized in space that ensures the synchronization of expression regulation (Rogozin, 2002; Llopis et al., 2010). WP_012855634.1 can likely play a role in the connection between central metabolism and post-transcriptional regulation of gene expression in *M. hominis*.

To summarize, the growth of *M. hominis* in the presence of thymidine leads to the downregulation of multiple proteins that enhance ribosome processivity or fidelity. We propose that this may lead to slower or less accurate translations in general. The latter may include pausing, stop-codon read-through, and mistranslation. These events may lead to general growth retardation. On the other hand, an increased level of chaperone ClpB rescues proteins from an aggregated state.

### Non-Cytoplasmic Proteins Are Significantly Changed Under Thymidine Condition

**Figure 6:**
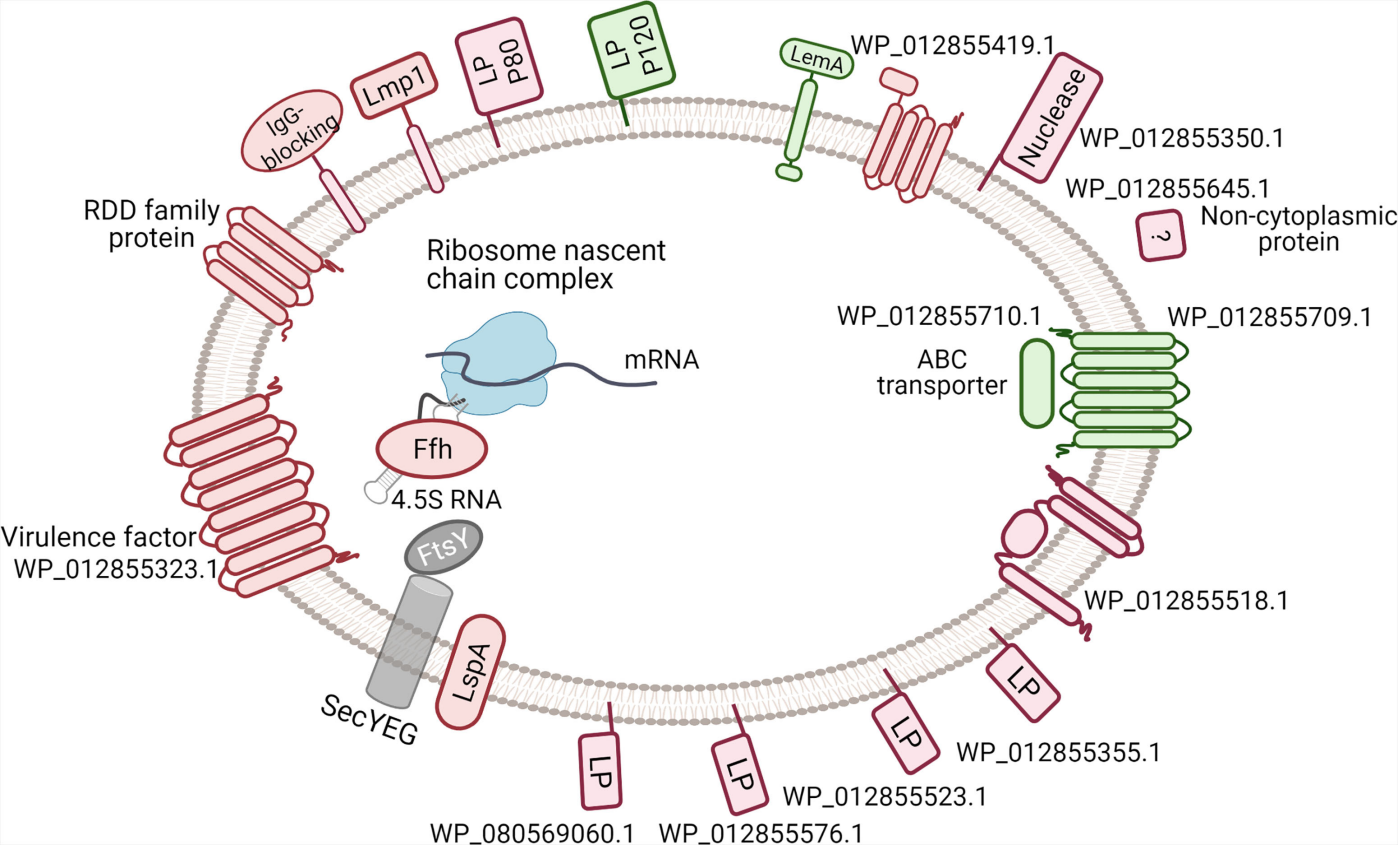
Difference in the membrane-associated proteins under thymidine conditions comparing with arginine conditions. The down-regulated proteins are indicated red, up-regulated - in green, unchanged - in grey. The data about signal peptides, Lipo-box, transmembrane helices and functional domains was used in protein diagramming. The SRP-mediated protein targeting is shown. LP - lipoprotein. Only two significantly changed transmembrane proteins are not shown: PotC and PepF. The corresponding full names of proteins are given in **Supplementary Tables S3**, **S4**.

In *M. hominis* grown on thymidine, we detected a reduced level of two proteins from the translocation system: signal recognition particle protein (Ffh) and signal peptidase II (LspA). Signal peptidase II (LspA) plays a crucial role in the subcellular localization and export of lipid-modified bacterial proteins (Vogeley et al., 2016). Moreover, signal peptidase I that is responsible for generating mature non-lipoproteins is not found in most Mycoplasma species (excluding avian pathogens), but some authors have observed its activity (Catrein et al., 2005). Ffh and 4.5S RNA form a signal recognition particle (SRP) that provides a translationally coupled mechanism of protein translocation (Akopian et al., 2013). Mycoplasmas are limited in the pathways of protein targeting and translocation. The change in the level of two important components of protein export prompted us to test the localization of up- and down-regulated proteins. We found membrane-associated proteins in the both groups. There were more of such proteins in the group of down-regulated proteins and the fold change value was greater. Most of the predicted membrane-associated proteins (**Figure 6**) do not have assigned annotations (**Supplementary Tables S3**, **S4**), but have lipobox, a conserved sequence at the C-region of the signal peptides with cysteine residue required for further modification and anchoring in the membrane (Kovacs-Simon et al., 2011) (**Supplementary Table S6**).

In bacteria, particularly pathogens, lipoproteins play key roles in adhesion to host cells and modulate immune responses (Christodoulides et al., 2018). The lipoprotein variations provide advantages of phenotypic plasticity of mycoplasmas, allowing survival and persistence within the host (van der Woude and Bäumler, 2004). The major lipoprotein Vaa did not change in our experiments. Among membrane-associated proteins, we discovered integral proteins that can be part of the signal transduction pathway, virulence, or transport. In the up-regulated group, hypothetical protein WP_012855709.1 has a primary structure similar to the ABC transporter permease, six transmembrane helices can be predicted (**Supplementary Figure S4**), and its neighbor WP_012855710.1 (upregulated too) is annotated as an ABC transporter ATP-binding protein, but it is localized in the cytoplasm. The level of conserved LemA protein (WP_012855649.1) was slightly increased on thymidine. This protein has a large internal fragment and a small extracellular amino terminus (**Supplementary Figure S5**). The molecular function of LemA remains unknown. Among the down-regulated proteins, lipid-anchored WP_012855350.1 unique for *M. hominis* and two Ureaplasma species membrane protein WP_012855645.1, decreased by 11- and 6-fold, respectively, in the thymidine condition. Recently, it was shown that protein WP_012855350.1 (gene name MHO_0730) is a surface-exposed nuclease that promotes neutrophil extracellular trap escape (Cacciotto et al., 2019). There were no functional domains in the WP_012855350 protein and only two coiled coil regions were predicted using the InterProScan software (**Supplementary Table S4**). Eight transmembrane helices containing protein WP_012855323.1, related to the YihY/virulence factor BrkB family, were also down-regulated on thymidine and integral membrane proteins WP_012855330.1 (Lmp1), WP_012855725.1 (RDD family protein), and WP_012855518.1(hypothetical protein). Changes in proteins associated with virulence suggest the importance of an energy source for *M. hominis* survival in an aggressive environment and spread in the host.

In our study, we have shown that the abundance of membrane-associated proteins changes during growth on thymidine. However, the functions of most proteins remain unclear. The role of surface and membrane proteins is enormous for a pathogen that loses its cell wall. In the future, to elucidate the mechanisms of pathogenicity and host-microbe communication, it will be necessary to conduct a deep analysis of the described proteins.

We described two major groups of proteomic changes for *M. hominis* grown on thymidine. As we demonstrated above, such cells had different susceptibility to antibiotics, especially to tetracycline. Decreased level of proteins for specifical modification of 16S rRNA could reduce tetracycline affinity to binding site (Schedlbauer et al., 2015; Grossman, 2016). An active efflux and reduced uptake of antibiotics is another mechanism of resistance (Reygaert, 2018). A changed level of ABC-transporters like PotC and WP_012855709.1 could influence drug availability but specificity of these proteins should be verified.

### Possible Modulators of Described Proteomic Effect

Under thymidine conditions, we detected an increased level of serine/threonine-protein phosphatase (WP_012855759.1), similar to PrpC (**Supplementary Figure S6**). In *Mycoplasma pneumonia*, as well as *M. hominis* and gram-positive bacteria, the *prpC* gene is downstream of *prkC* that encodes another serine/threonine protein kinase. Inactivation of *prkC* in *M. pneumonia* leads to a non-adherent, low-cytotoxicity phenotype. PrkC-dependent phosphorylation of large cytadherence proteins is required for the stability of these complexes (Schmidl et al., 2010). PrpC is thought to be an antagonist of PrkC (Gaidenko et al., 2002). Therefore, the decreased levels of some membrane-associated proteins can potentially be explained by a reduction in PrpC abundance. Moreover, the stationary-phase cell density of the *prpC*-null mutant *Bacillus subtilis* was significantly higher than that of the wild type (Gaidenko et al., 2002); therefore, PrpC could be a participant in cell-cell communication. It seems surprising that the abundance of many lipoproteins decreases and only a few increases in *M. hominis* grown on thymidine, while biofilm formation is induced under such conditions. For *Mycoplasma bovis*, it has been shown that different patterns of variable membrane surface lipoproteins may display different adhesion capabilities, resulting in the ability to form biofilms (McAuliffe et al., 2006; Bürki et al., 2015).

The abundance of DUF448 domain-containing protein WP_012855794.1 decreased four-fold in *M. hominis* grown on thymidine compared to arginine. This protein is similar to YlxR (RulR) from *B. subtilis.* YlxR is widely conserved in eubacteria, but its function remains elusive. Recent studies on *B. subtilis* showed that YlxR is a nucleoid-associated protein (Ogura and Kanesaki, 2018); it is involved in glucose-responsive metabolic changes (Ogura et al., 2019). *M. hominis* and some other members of class Mollicutes have lost some enzymes for glucose utilization (Pereyre et al., 2009). We suppose that YlxR is capable of performing a regulatory function in response to the energy state of the cell regardless of the availability of glucose.

## Conclusion

In summary, the growth of *M. hominis* on thymidine-supplemented medium led to a significant difference in growth rate compared to bacteria cultivated on arginine. This slow-growth phenotype of bacteria demonstrates reduced susceptibility to some antibiotics and an increased extent of biofilm formation. Our comparative proteomic analysis of *M. hominis* grown on different energy sources confirmed metabolic rearrangement and allowed us to identify two major groups of differentially expressed proteins: translation-related and membrane-associated proteins, and their translocation. The first one is responsible for a slower or less accurate translation, and the second one is the variation of immunogenic proteins on the cell surface and virulence. These results may help uncover the molecular basis of the slow bacterial growth phenotype and improve the strategies for developing novel antimicrobial agents for combating mycoplasmosis. Future studies should focus on identifying the effector molecules that provide signal transduction and regulation of *M. hominis* switching to a slow-growth phenotype. In parallel, the elucidation of the exact function of membrane-associated proteins could cover a blind spot in the physiology and pathogenicity of genome-reduced bacteria.

## Data Availability Statement

The datasets presented in this study can be found in online repositories. The names of the repository/repositories and accession number(s) can be found in the article/**Supplementary Material**.

## Author Contributions

DE contributed to conception and design of the study, worked with nucleic acid, wrote the manuscript. TS contributed to conception and design of the study, analysed proteomic data. VL carried out cultivation of bacteria. MG performed biofilm assay. SK and RZ performed mass-spectrometry analysis. GF contributed to conception of the study, wrote section of the manuscript. OP contributed to conception of the study, prepared samples for proteomic analysis. All authors contributed to manuscript revision, read, and approved the submitted version.

## Funding

This work was supported by the Russian Science Foundation (project number 19-75-10124).

## Conflict of Interest

The authors declare that the research was conducted in the absence of any commercial or financial relationships that could be construed as a potential conflict of interest.

## Publisher’s Note

All claims expressed in this article are solely those of the authors and do not necessarily represent those of their affiliated organizations, or those of the publisher, the editors and the reviewers. Any product that may be evaluated in this article, or claim that may be made by its manufacturer, is not guaranteed or endorsed by the publisher.
