# Supplementary material for: Molecular Basis of the Slow Growth of Mycoplasma hominis on Different Energy Sources
Source: Front Cell Infect Microbiol. 2022 Jul 7;12:918557. doi: 10.3389/fcimb.2022.918557 (PMC9301678; doi:10.3389/fcimb.2022.918557)
Supplement: Supplementary file 1 [file Image_1.pdf]

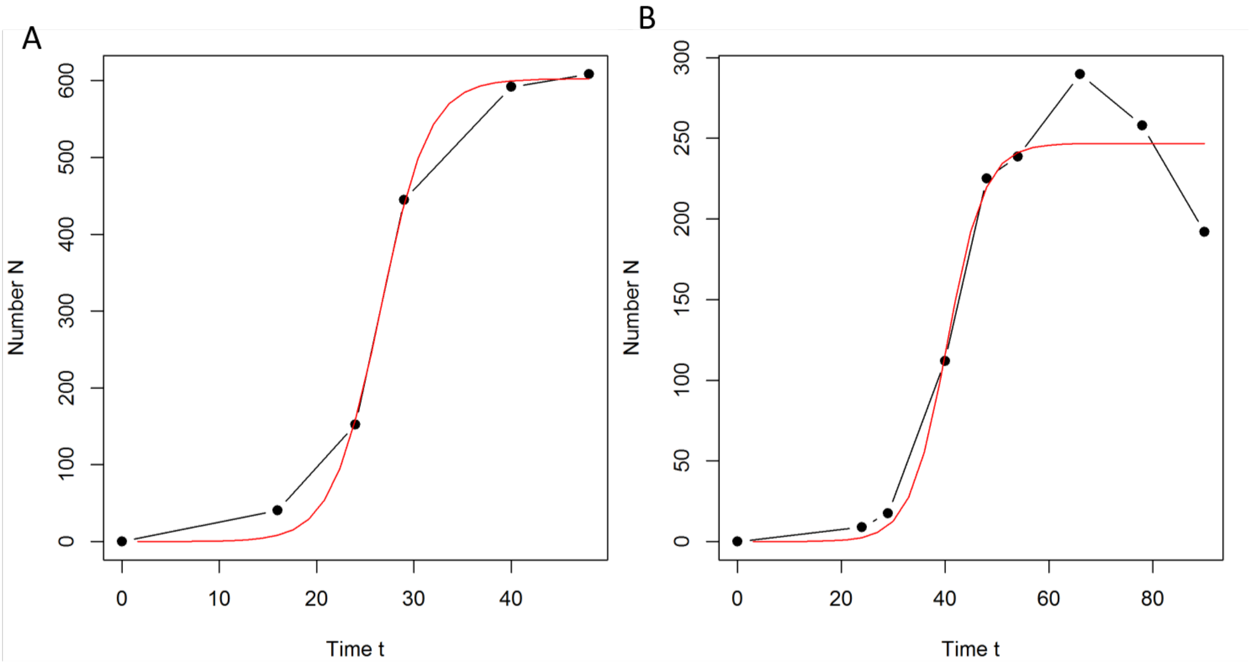

Fig. S1. The growth curves of *M. hominis* on medium supplemented with arginine (A) or thymidine (B). Fitted growth curve data by the standard logistic equation, the value of arguments can be found in Supplementary Table S1.

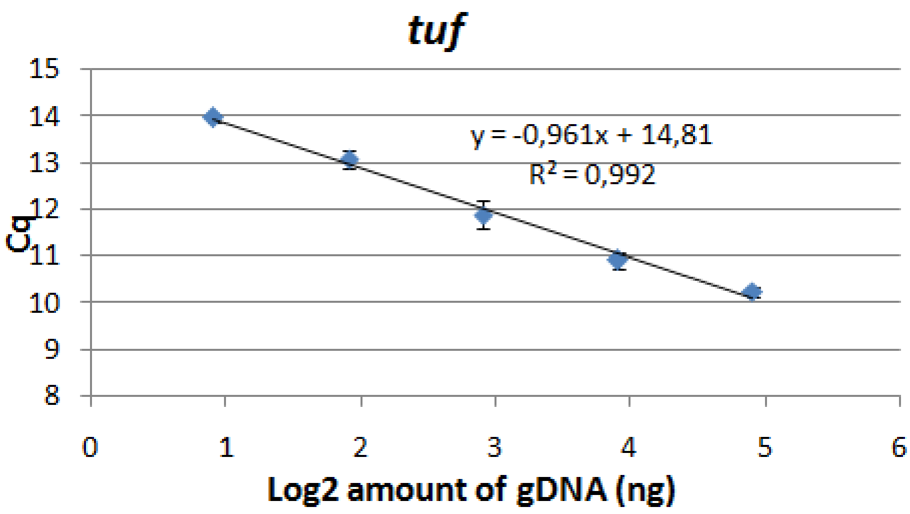

Fig. S2. Calculation of the amplification efficiency from a qPCR experiment. Genomic DNA of *Mycoplasma hominis H-34* was isolated and concentration was determined using Qubit 2.0. Standard curve was plotted for five twofold serial dilutions of gDNA. The slope of the standard curve was used to calculate the PCR efficiency. For *tuf* primer pair, PCR efficiency was 105%. PCR primer efficiency was calculated as Efficiency (%) = $(2^{-1/\text{slope value}}-1)*100$

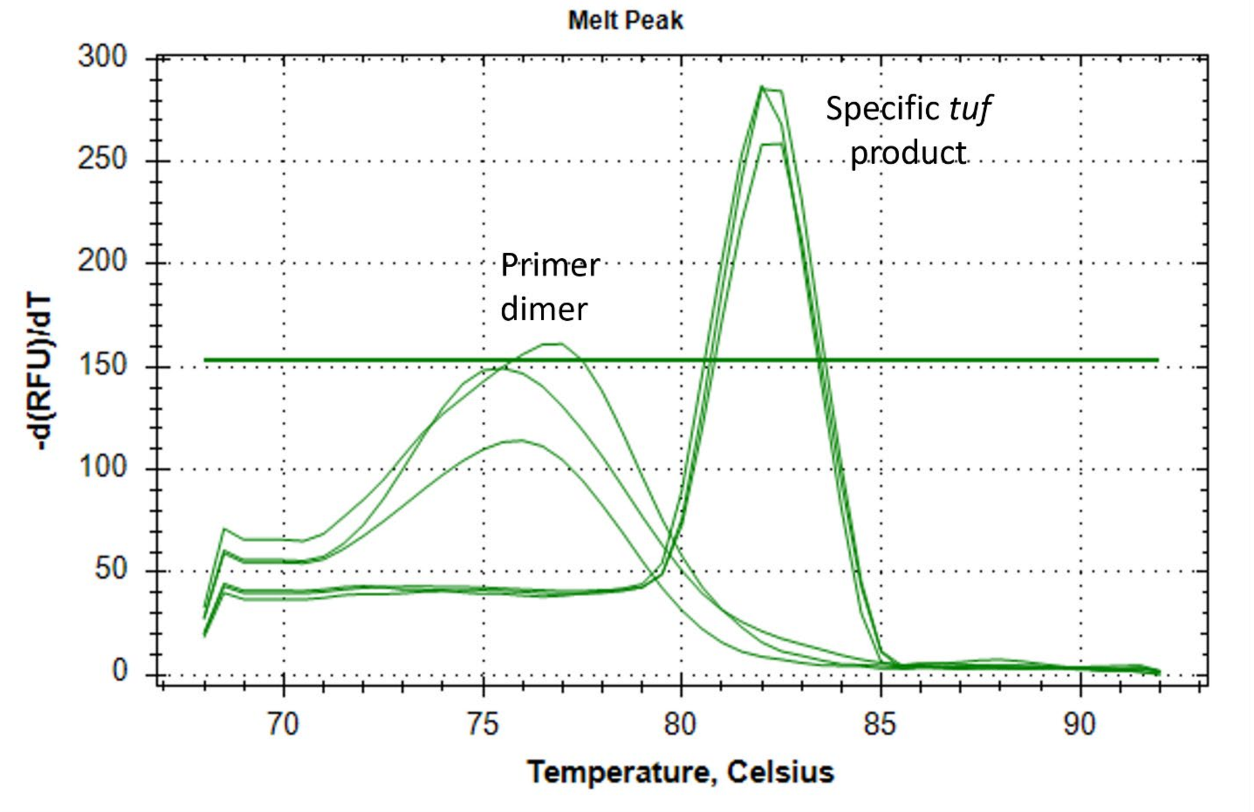

Fig. S3. Melting curves analysis of the *tuf* PCR products. The left peaks are corresponded to products from three negative control samples, while the right - from the three arginine-cultivated mycoplasma samples under the highest tetracycline concentration.

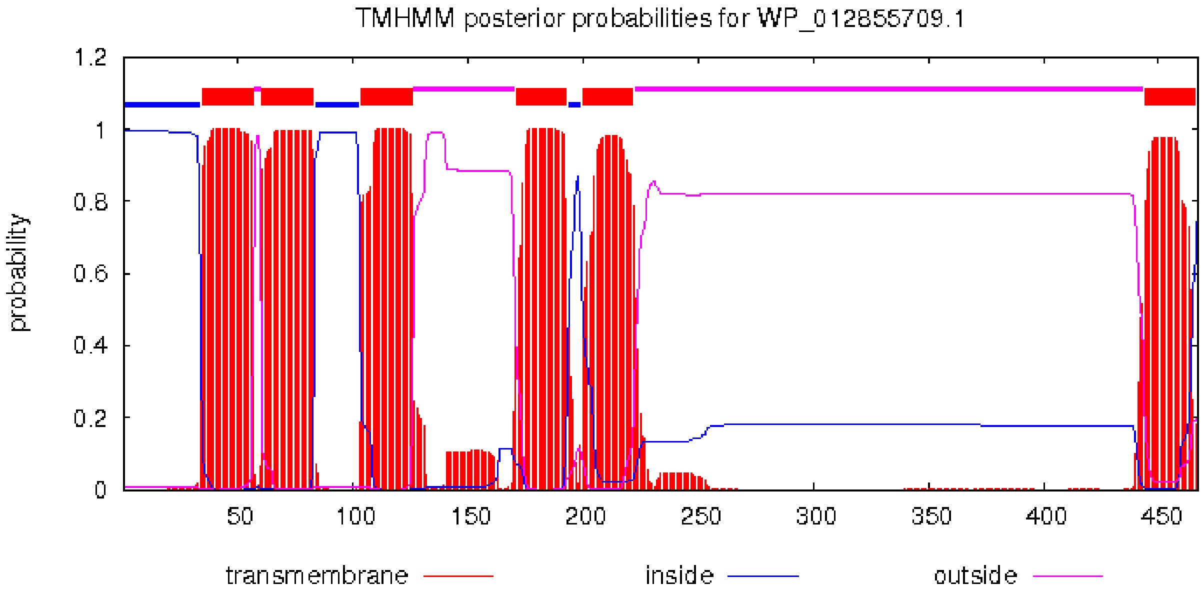

Fig. S4. Results of transmembrane helices prediction by TMHMM for protein WP_012855709.1. Red bars indicate transmembrane domains, blue lines indicate

intracellular loops and magenta lines indicate extracellular loops. Six transmembrane helices can be predicted for WP_012855709.1.

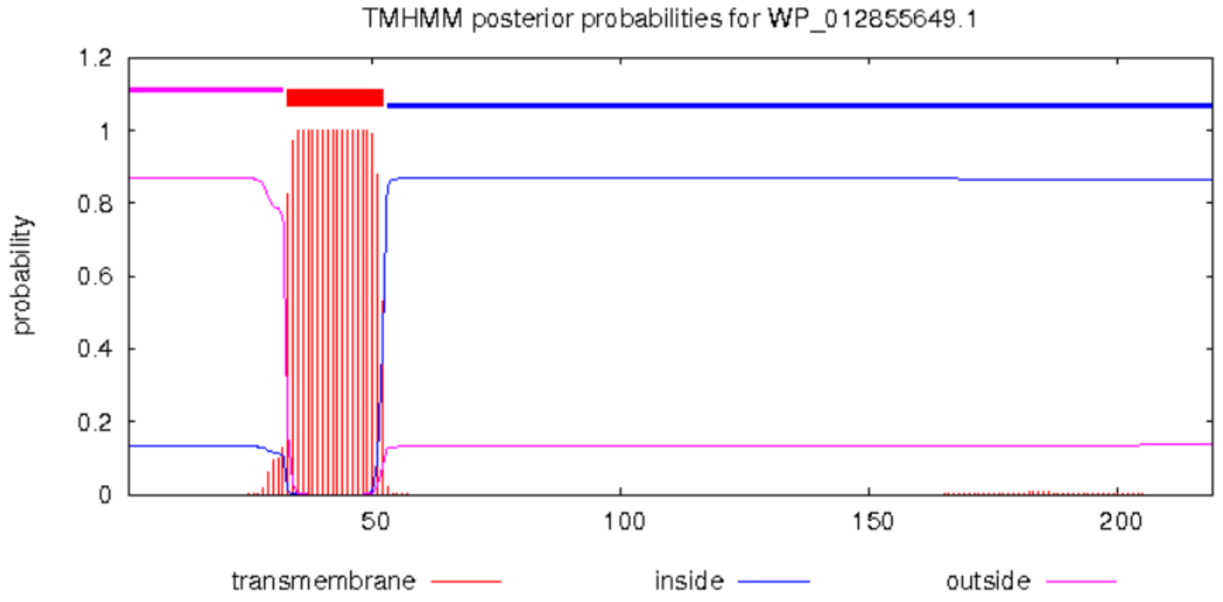

Fig. S5. Results of transmembrane helices prediction by TMHMM for protein WP_012855649.1 (LemA). Red bars indicate transmembrane domains, blue lines indicate intracellular loops and magenta lines indicate extracellular loops. Protein LemA has a large inside fragment and small extracellular amino terminus.

```
PPM1A_HUMAN         MGAFLDKPKMEKHNAQGQGNGLRYGLSSMQG-WRVEMEDAHTAVIGLPSGLESWSFFAVY  59
WP_012855759.1      --------------------MVDFCIASDQGKFRKENQDRAI-----VVNGEYWTLAALC  35
PRPC_BACSU          ---------------------MLTALKTDTGKIRQHNEDDA----GIFKGKDEFILAVVA  35
                                         :   : :  *  * .    :        . : : : .:

PPM1A_HUMAN         DGHAGSQVAKYCCEHLLDHITNN--QDFKGSAGAPSVENVKNGIRTGFLEIDEHMRVMSE  117
WP_012855759.1      DGMGGHVGGSQCSTLSTNSIKQYFLDTFPQKLECSDKKNVSKWFNNAISFIKEALVNYAK  95
PRPC_BACSU          DGMGGHLAGDVASKMAVKAMGEKWNEAETI---PTAPSECEKWLIEQILSVNSKIYDHAQ  92
                    ** .*   .. ..    . : :   :           .: .: :   :  :.. :   ::

PPM1A_HUMAN         KKHGADRSGSTAVGVLISPQ--HTYFINCGDSRGLLCRNRKVHFFTQDHKPSNPLEKERI  175
WP_012855759.1      EYTEFEDMGTTMVVALIFNANGLAYVFNIGDSRLYAYNG-LLYQITEDQNYLYQLMREFN  154
PRPC_BACSU          AHEECQGMGTTIVCALFTGK--TVSVAHIGDSRCYLLQDDDFVQVTEDHSLVNELVRTGE  150
                         :  *:* * .*:      . . : ****    ..  .  .*:*:.    * :

PPM1A_HUMAN         QNAGGSVMIQRVNGSLAVSRALGDFDYKCVHGKGPTEQLVSPEPEVHDIERSEEDDQFII  235
WP_012855759.1      LTYEE--------------AALDPNSYKLISCLGPNK-KTNCQSFFIS---QKSAVKYYL  196
PRPC_BACSU          ISRED--------------AEHHPRKNVLTKALGTDQ-LVSIDTRSFD---I-EPGDKLL  191
                     .                       .       *  :  .. :    .     .  .  :

PPM1A_HUMAN         LACDGIWDVMGNEELCDFVRSRLEVTDDLEKVCNEVVDTCLYKGSRDNMSVILICFPNAP  295
WP_012855759.1      LTSDGLHDYVSKPIIETVLQTNKSL----KDKLNLLIKYAKKNLSKDNITGILVGLKNE-  251
PRPC_BACSU          LCSDGLTNKVEGTELKDILQSDSAP----QEKVNLLVDKANQNGGEDNITAVLLELALQV  247
                    * .**: : :    :  .:::        :.  * ::. .  : ..**:: :*: :

PPM1A_HUMAN         KVSPEAVKKEAELDKYLECRVEEIIKKQGEGVPDLVHVMRTLASENIPSLPPGGELASKR  355
WP_012855759.1      ------------------------------------------------------------  251
PRPC_BACSU          EEGEDQC-----------------------------------------------------  254

PPM1A_HUMAN         NVIEAVYNRLNPYKNDDTDSTSTDDMW    382
WP_012855759.1      ---------------------------    251
PRPC_BACSU          ---------------------------    254
```

Fig. S6. Sequence alignment of protein phosphatase 2C from *Homo sapience*, *Bacillus subtilis* and *Mycoplasma hominis*. Residues binding the metal ions and phosphate ions are indicated by yellow and green, respectively. The position of sites from [Das, A. K., Helps, N. R., Cohen, P. T., & Barford, D. (1996). Crystal structure of the protein serine/threonine phosphatase 2C at 2.0 A resolution. *The EMBO journal*, *15*(24), 6798–6809.]
